# Exposure to Light of the Abaxial versus Adaxial Side of Detached *Kalanchoë blossfeldiana* Leaves Affects Anthocyanin Content and Composition Differently

**DOI:** 10.3390/ijms25052875

**Published:** 2024-03-01

**Authors:** Wiesław Wiczkowski, Marian Saniewski, Agnieszka Marasek-Ciołakowska, Justyna Góraj-Koniarska, Joanna Mitrus, Marcin Horbowicz

**Affiliations:** 1 Department of Chemistry and Biodynamics of Food, Institute of Animal Reproduction and Food Research of the Polish Academy of Sciences, Tuwima 10, 10-748 Olsztyn, Poland; w.wiczkowski@pan.olsztyn.pl; 2 The National Institute of Horticultural Research, Konstytucji 3 Maja 1/3, 96-100 Skierniewice, Polandagnieszka.marasek@inhort.pl (A.M.-C.); justyna.goraj@inhort.pl (J.G.-K.); 3 Institute of Biological Sciences, University of Siedlce, Prusa 14, 08-110 Siedlce, Poland; joanna.mitrus@uph.edu.pl; 4 Department of Plant Physiology, Genetics and Biotechnology, University of Warmia and Mazury, Oczapowskiego 1A, 10-719 Olsztyn, Poland

**Keywords:** *Kalanchoë blossfeldiana*, leaf, storage, anthocyanin, profile, abaxial side, adaxial side, methyl jasmonate

## Abstract

The accumulation and composition of anthocyanins in leaves of *Kalanchoë blossfeldiana*, detached and kept for five days under natural light conditions, were investigated. The presence of fifteen derivatives of cyanidin, petunidin, and delphinidin was found. Changes in the content of each anthocyanin in the leaves before and after exposure to light on the abaxial (naturally upper) and adaxial (naturally lower) sides of the leaves were compared. When the adaxial side was exposed to light, the anthocyanin contents of the leaves did not change. In contrast, when the abaxial side of detached leaves was exposed to light, there was enhanced accumulation of delphinidin-rhamnoside-glucoside, cyanidin-rhamnoside-glucoside, cyanidin-glucoside-glucoside, and two unknown derivatives of petunidin and delphinidin. Application of methyl jasmonate (JA-Me) on the abaxial side exposed to light inhibited the accumulation of these anthocyanins. This effect could probably be due to the presence of these anthocyanins in the epidermal cells of *K. blossfeldiana* leaves and was visible in the microscopic view of its cross-section. These anthocyanins were directly exposed to JA-Me, leading to inhibition of their formation and/or accumulation. The lack of significant effects of JA-Me on anthocyanin mono- and tri-glycosides may indicate that they are mainly present in the mesophyll tissue of the leaf.

## 1. Introduction

Hundreds of structurally distinct anthocyanins, which are derivatives of several anthocyanidins, are produced in the tissues of plants, but it is not known what functions each type has. However, the high metabolic cost of creating anthocyanidin derivatives means that they can play important though varied roles [1,2,3,4].

The physiological, biochemical, and molecular mechanisms underlying the effects of various factors on anthocyanin levels in plants have been widely discussed [5,6,7,8,9]. Anthocyanins present in flowers are responsible for their color, thus playing an important role in attracting insects and, consequently, in pollination and seed dispersal [10]. In leaves, anthocyanins accumulate in response to nutrient deficiencies, environmental conditions, as well as damage, or as a defense against pests, pathogenic fungi, or drought stress [7,11,12,13,14,15,16,17]. Plant leaves turn red or purple under stressful conditions such as low temperatures, high light levels, or nutrient deficiency [18]. An interesting and comprehensive review describes the role of anthocyanins in acclimating plants to nutrient stress caused by deficiencies of nitrogen, magnesium, phosphorus, sulfur, and other minerals [18]. In response to drought stress, anthocyanins act as both osmoregulators and antioxidants, whereby the antioxidant potential of anthocyanins depends on the chemical structure of these pigments. In response to drought stress, anthocyanins act as both osmoregulators and antioxidants [5,15,18].

However, the most recognized factor affecting anthocyanin content is light, including both its intensity and spectral range [6,12,19]. Anthocyanin concentration in leaves increases under excessive light conditions [20,21]. An increase in the expression of flavonoid pathway genes accompanied by an increase in the content of phenolic compounds, including anthocyanins, was observed in blueberry (*Vaccinium myrtillus*) leaves grown in direct sunlight [22]. Anthocyanins act as filters of light, especially from the green–orange regions of the spectrum, and dissipate the absorbed energy as heat [4,13,23,24]. Since anthocyanins accumulate in the epidermal tissues of leaves, they may contribute to protecting photosynthetic mesophyll tissues from UV-B [4,25,26,27,28]. It has been shown that UV-B induces a decrease in anthocyanidin reductase activity, resulting in an increase in anthocyanin biosynthesis [29]. Among the anthocyanins, their glycosides have little ability to absorb UV light, but acyl derivatives absorb both visible and UV light [4,25].

The detachment of leaves from the plant causes partial wounding of the petioles and activates defense factors [30]. Ethylene plays an important role in the wound response, which, in combination with jasmonates and ABA, probably mediates local reactions at the wound site [31,32,33,34]. Ethylene is quickly synthesized and accumulates in response to wounding, then diffuses into surrounding tissues and induces anthocyanin accumulation, as demonstrated in many studies [35]. Wounding or treatment with methyl jasmonate, and the associated increase in ethylene levels, triggers the plant’s defenses by accumulating protective metabolites [36]. According to Shin et al. [37] anthocyanins accumulated in leaf explants of *Arabidopsis thaliana*, but not near the wound site. The authors also showed that anthocyanin accumulation at sites without wounds was required for drought resistance in leaf explants.

It is interesting that treatment of *K. blossfeldiana* plants with gaseous ethylene did not induce leaf abscission and anthocyanin accumulation, but treatment of these plants with a methyl jasmonate solution caused complete leaf abscission and stimulated ethylene formation [38].

Changes found in anthocyanin profiles in *Arabidopsis thaliana* plants exposed to different abiotic stresses indicate that not all anthocyanins have the same function [2,3]. Authors showed that under stress conditions, *Arabidopsis* not only often accumulates significantly higher total anthocyanin contents but also promotes the accumulation of individual anthocyanins. This suggests that various anthocyanins have different functions under specific stress conditions [2,3,39].

*Kalanchoë blossfeldiana* belongs to Crassulacean acid metabolism (CAM) plants and short-day plants. Under long-day conditions, its leaves accumulate a much higher content of phenolic compounds compared to leaves growing under short-day conditions [29]. Leaves of *K. blossfeldiana* become red during the flowering stage, especially at the apical end on the underside, and the major pigment is cyanidin-3-glucoside (chrysanthemin) with minor amounts of cyanidin-3,5-diglucoside (cyanin) [40]. According to the authors, the major anthocyanin of the *K. blossfeldiana* flowers was cyanin with minor amounts of chrysanthemin and probably pelargonidin-3,5-diglucoside (pelargonin) [40]. Later, it was found that flowers of *K. blossfeldiana* cultivars contain diglucosides of pelargonidin, cyanidin, peonidin, delphinidin, petunidin, and malvidin [41].

Saniewski et al. [42] reported that methyl jasmonate (JA-Me) applied in lanolin paste stimulated the formation and accumulation of anthocyanins in the shoots of young *K. blossfeldiana* plants. Additionally, Góraj-Koniarska et al. [43] demonstrated that JA-Me also stimulated anthocyanin accumulation in *K. blossfeldiana* roots that were exposed to natural light in the greenhouse. Subsequently, our previous study showed that in detached *K. blossfeldiana* leaves that were kept in an inverted position for five days, higher anthocyanin accumulation occurred on the abaxial side [44]. Moreover, the application of JA-Me significantly inhibited the anthocyanin accumulation induced on the abaxial side. Since the total anthocyanin content was analyzed, this study attempts to assess whether and how the above-mentioned factors affect the accumulation of particular types of anthocyanin in *K. blossfeldiana* leaves.

## 2. Results and Discussion

### 2.1. Anthocyanin Profiles and Their Changes in K. blossfeldiana Leaves

**Figure 1:**
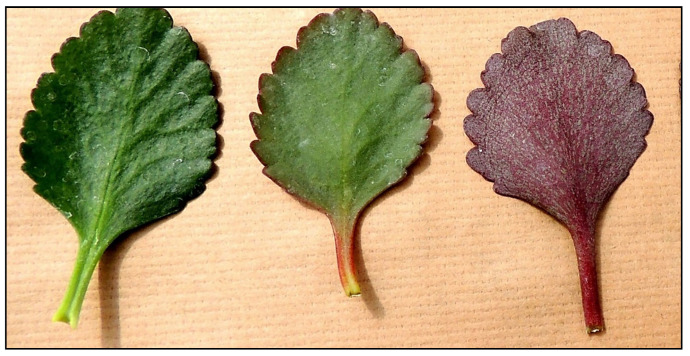
Leaves of *K. blossfeldiana* leaves after detaching from the plant (**left**); after keeping the adaxial side exposed to ambient light for five days (**center**), and after keeping the abaxial side exposed to ambient light for five days (**right**).

Anthocyanin accumulation in plants under abiotic stress is well-documented [2,3,8,45]. In our previous paper, it was shown that in *K. blossfeldiana* leaves detached from the plant and maintained for five days in an inverted position, higher total anthocyanin contents accumulate on the side exposed to light (abaxial) than on the adaxial side, as demonstrated in Figure 1 [44]. During the mentioned study, analyses of the total anthocyanin content using a commonly used spectrophotometric method were performed. The present study was undertaken to assess whether all types of anthocyanins in *K. blossfeldiana* leaf tissue react in similar or different ways.

Using the micro HPLC/MS/MS-TOF method, the presence of seven cyanidin derivatives, six delphinidin derivatives, and two petunidin derivatives was confirmed in *K. blossfeldiana* leaf tissue (Table 1). Of these fifteen anthocyanins, there were two mono-glycosides, four di- glycosides, and three triglycosides, while the other three were glycosides acylated with phenolic acids. In addition, two petunidin derivatives, one delphinidin, and one cyanidin derivative were found that had not been identified previously. The method for identifying derivatives used the retention times of their peaks, the values of parent ion, and the fragmentation spectra obtained, and was based on previous papers [46]. The results obtained by contemporary methods only to a small extent confirm the results of Neyland et al. [40], who detected in the leaves of this plant at the flowering stage the presence of only cyanidin-3-glucoside as the main anthocyanin and small contents of cyanidin-3,5-diglucoside. Previously, in the roots of *K. blossfeldiana*, eight anthocyanins were found, and quantitatively, the main one was cyanidin 3,5-di-glucoside [43].

**Figure 2:**
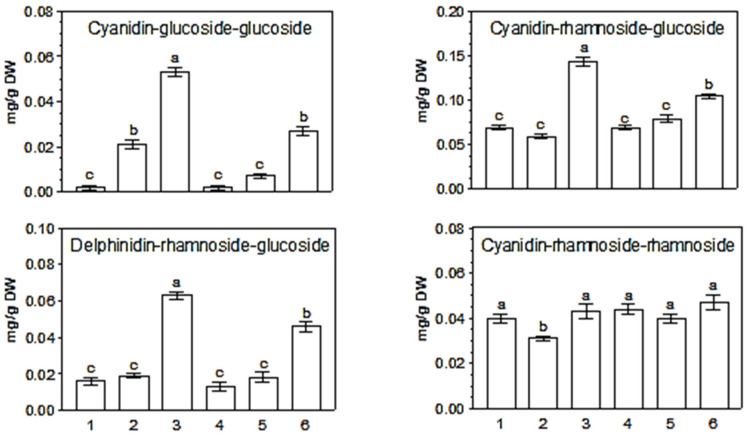
Content of anthocyanin di-glycosides in *K. blossfeldiana* leaves on the plant and after detachment and five-day storage in natural (adaxial side exposed to light) or inverted (abaxial side exposed to light) positions. Description of results: 1—Large leaves on plant; 2—Large leaves detached from the plant and stored in their natural position; 3—Large leaves detached from the plant and stored in an inverted position; 4—Small leaves on plant; 5—Small leaves detached from the plant and stored in their natural position; 6—Small leaves detached from the plant and stored in an inverted position. Bar results followed by the same letters do not differ significantly at *p* = 0.05.

**Figure 3:**
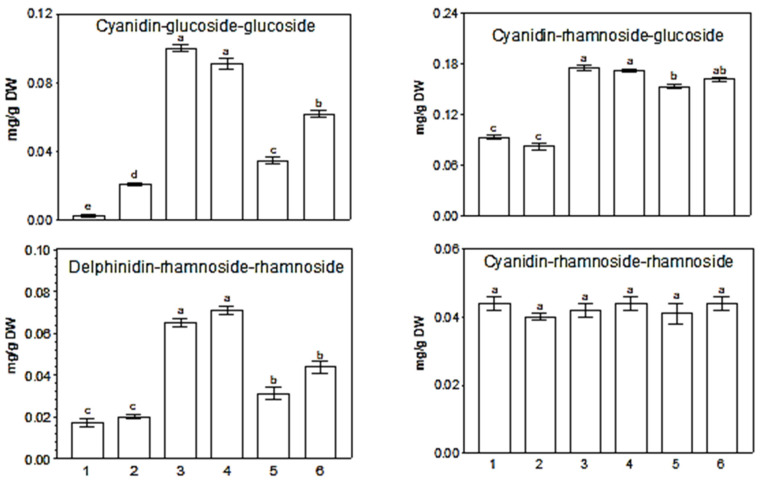
Content of anthocyanin diglycosides in leaves of *K. blossfeldiana* on the plant, after detachment and 5-day storage in natural position (adaxial side exposed to light) or stored in inverted position (abaxial side exposed to light) and treated with methyl jasmonate (JA-Me) in lanolin. Description of results: 1—Leaves on plant; 2—Leaves detached from the plant and stored in their natural position; 3—Leaves detached from the plant and stored in an inverted position; 4—Leaves detached from the plant, treated with lanoline and stored in an inverted position; 5—Leaves detached from the plant, treated with 0.5% JA-Me in lanolin and stored in an inverted position; 6—Leaves detached from the plant, treated with 0.1% JA-Me in lanoline and stored in an inverted position. Bar results followed by the same letters do not differ significantly at *p* = 0.05.

In leaves detached from the plant *of K. blossffeldiana* and stored for five days naturally (adaxial side towards the light), the content of most anthocyanins decreased (Figure 2 and Figure 3; Table 2 and Table 3). In contrast, five-day storage of leaves with the lower (abaxial) side towards the light resulted in a 2- to 3-fold increase in the contents of delphinidin rhamnoside-glucoside, cyanidin rhamnoside-glucoside, and cyanidin di-glucoside. These anthocyanins are probably related to the red color that appears on the abaxial side of the leaves, as shown in the pictures in our previous paper [44]. They are probably transferred from the deeper layers of the leaf to the marginal layers as a result of exposing the abaxial side of the leaf to light.

The content of mono- and tri-glycosides in *K. blossfeldiana* leaves on the plant and after detachment and five-day storage in natural (adaxial side exposed to light) or inverted (abaxial side exposed to light) positions were similar or lower (Table 2 and Table 3). Since these anthocyanins were also found in leaves on the plant that do not have a red coloration, this may suggest that they occur in the inner layers of the leaf.

Calculated coefficients of *r*-Pearson’s correlation (0.981) and *rho*-Spearman’s correlation (0.983) between tri-glycosides and di-glycosides content were highly significant. Similarly, such coefficients between cyanidin-rhamnoside-glucoside and cyanidin-rhamnoside-rhamnoside-glucoside were highly significant at 0.864 and 0.983, respectively. These data indicate that the accumulations of the two types of anthocyanins are closely related.

Particularly important are changes in the content of the unknown petunidin derivative 1, which is quantitatively dominant in *K. blossfeldiana* leaves (Table 2 and Table 3). However, these changes are not consistent, since in Experiment 2, the content of this compound was higher in leaves kept in the inverted position than in the normal position, while in Experiment 1, the results were similar for both positions. A similar situation was observed for unknown petunidin 2 and cyanidin derivatives. The reasons for this situation are unknown. It seems that they may be caused by factors related to the cultivation of the plant, such as light conditions.

It is interesting that methyl jasmonate (JA-Me) applied in lanolin paste on the abaxial part of the leaves maintained in an inverted position inhibited accumulation of antocyanin diglycosides: delphinidin-rhamnoside-glucoside, cyanidin-glucoside-glucoside, and cyanidin-rhamnoside-glucoside (Figure 3). In contrast to the leaves, application of JA-Me to the stem of young *K. blossfeldiana* plants caused a marked accumulation of total anthocyanins in this organ [42]. Anthocyanins were accumulated in the main shoot and in lateral shoots and petioles, both below and above the treated sections. However, when the leaves were removed, almost no anthocyanin formation was observed. It was later found that JA-Me substantially increased anthocyanin content in the roots of intact *K. blossfeldiana* plants under natural light conditions [43]. This means that distinct organs of *K. blossfeldiana* show varied responses to the action of JA-Me.

The JA-Me not affect the content of mono-glycosides and tri-glycosides of cyanidin and delphinidin (Table 3). The influence of JA-Me on anthocyanin diglycosides may be due to their presence in the peripheral epidermal cells of *K. blossfeldiana*, which are directly exposed to JA-Me, leading to inhibition of their formation and/or accumulation. The lack of a significant effect of JA-Ma on mono-glycosides and tri-glycosides may, in turn, indicate that these anthocyanins are present in the mesophyll parts of the leaf tissues.

The various responses of individual anthocyanins to wounding and light stress during the storage of *K. blossfeldiana* leaves confirm information previously published by Kovinich et al. [2,3]. There was an increased accumulation of certain diglycosides in the epidermis in response to stresses caused by leaf storage conditions. This effect is probably a result of the action of ethylene, jasmonates, and/or other phytohormones in response to the stresses that occurred.

### 2.2. Histological Observations of K. blossfeldiana Leaves

**Figure 4:**
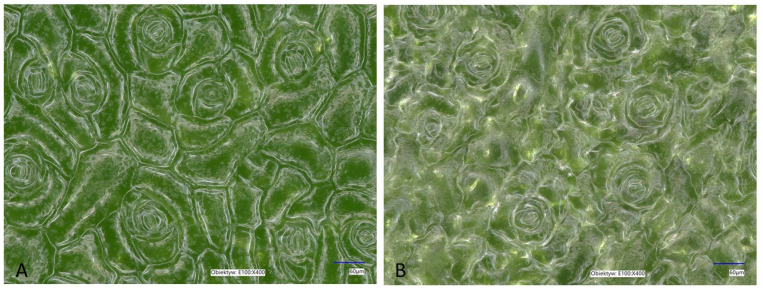
Optical microphotographs of the leaf surface of *K. blossfeldiana.* (**A**) Adaxial epidermis, (**B**) abaxial epidermis.

The reason for the different response involving increased accumulation of some anthocyanins on the abaxial side during the maintenance of leaves in the inverted position may be due to morphological differences between both sides of the leaf of *K. blossfeldiana*. In leaves of this species, the adaxial epidermis is made up of polyhedral (hexagonal and pentagonal) cells, while on the abaxial epidermis, the cells have folded walls and there are fewer stomata, and cells of the adaxial epidermis are larger than those of the abaxial epidermis [47]. According to this study, the cell lengths in the adaxial epidermis ranged from 132 to 225 nm, while the widths ranged from 62 to 100 nm. In comparison, the lengths and widths of abaxial epidermis cells ranged from 125 to 205 and 55 to 90 nm, respectively. Earlier, Jeong et al. [48] demonstrated that the density of stomata on the abaxial surface of *K. blossfeldiana* leaves was higher compared to the adaxial surface. Also, the leaf blades of *Kalanchoë daigremontiana* are bifacial and amphistomatic with an abaxial epidermis containing about twice as many stomata complexes as the adaxial epidermis [49]. Therefore, the effect of light on the abaxial and adaxial sides may also be dissimilar, due to differences in the structure of both sides. As a result, in response to light, there was an accumulation of some anthocyanins, such as diglycosides of delphinidin and cyanidin, in the abaxial tissue. However, in our study, the density of stomata in *K. blossfeldiana* leaves was similar in the adaxial and abaxial epidermis and was 21.8 ± 1.64 and 22.6 ± 1.82 per mm^2^, respectively (Figure 4). Also, the length of the stomata was similar for adaxial and abaxial epidermis: 44.09 ± 4.3 µm in the adaxial epidermis and 45.71 ± 4.1 µm in the abaxial epidermis. Thus, it seems that the differences in the anatomical structure of the two sides of the leaf were not the reason for the redness of the abaxial side caused by the accumulation of anthocyanins. It is clear, however, that the green color intensity of the adaxial epidermis is lower than that of the abaxial epidermis. Also, there are slight differences in the structure of the two epidermises.

**Figure 5:**
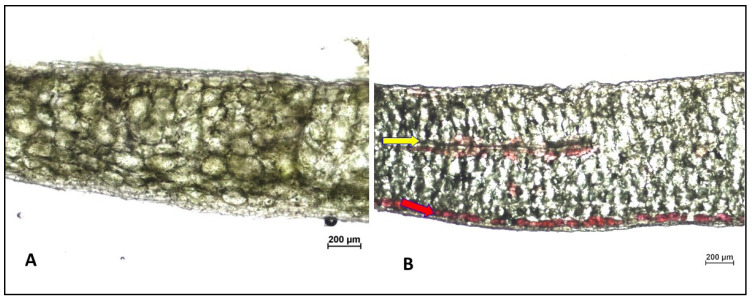
Microscopic cross-sectional pictures of a *K. blossfeldiana* leaf. (**A**) Leaf after detachment; (**B**) leaf kept for five days in an inverted position (abaxial side on light). The red arrow indicates the accumulation of anthocyanins under the epidermis of the abaxial side, while the yellow arrow indicates the accumulation of anthocyanins in mesophyll tissue (inside the leaf).

In general, secondary metabolites are asymmetrically distributed in the leaf tissue. In maize leaves, flavonoids are mainly located in the upper (adaxial) epidermis [50]. A higher rutin content in the adaxial epidermis than in the abaxial epidermis has been reported for common buckwheat (*Fagopyrum esculentum*) cotyledons as well [51]. Li et al. [52] studied spatial metabolomics in *Ginkgo biloba* leaves and documented that the distribution of different flavonoid classes was localized in both epidermises but with higher abundance in the adaxial epidermis. In these studies, analyses were performed for leaves in their natural position on the plant. Our present results indicate that a different response occurs in a leaf detached from a plant whose adaxial side was exposed to light. A microscopic cross-section of a *K. blossfeldiana* leaf shows that anthocyanins accumulate in two sites, under the epidermis and in the deeper layers of mesophyll cells (Figure 5). This may suggest that under the epidermis of the abaxial side, there are anthocyanins accumulated as a result of exposure of this side to light, mainly di-glycosides of cyanidin and delphinidin. However, it is likely that these changes found in detached leaves of *K. blossffeldiana* are the result of a few combined factors.

The detachment of a leaf from a plant shoot causes damage to the petiole, followed by mineral deficiencies, and results in drought stress and/or tissue desiccation. In addition, exposure of the abaxial side to light further stimulates red coloration and affects the profile of anthocyanins. The effects of a variety of factors on anthocyanin accumulation in plants have been described in detail in recently published reviews [10,18].

Permanent red–purple coloration is commonly observed in the abaxial epidermis of leaves of tropical plants growing in deeply shaded understory [53]. However, the ecophysiological role of abaxial anthocyanins in perennial understory plants from temperate deciduous forests is little known [54,55,56]. Environmental stress caused by inverting the leaves so that their abaxial surfaces are exposed to strong light causes them to redden as a result of anthocyanin accumulation [57]. The photoprotection hypothesis is currently the best-supported explanation for abaxial reddening to suppress excess light falling on the surface of inverted leaves or passing through adaxial cell layers [58].

It is known that sucrose can induce anthocyanin accumulation in plant tissues. Momose and Ozeki [59] showed that in detached leaves of the aquatic plant *Egeria densa* placed in water, there was chlorophyll degradation, and detached leaves turned yellow, but when incubated in 0.1 M sucrose, there was chlorophyll degradation and induction of anthocyanin accumulation.

Results presented in our previous paper showed that that sucrose content is much higher in leaves kept on the abaxial side to light than in the normal side (adaxial) [43]. Therefore, such a response can be an additional reason for enhanced anthocyanin accumulation in leaves with the abaxial side exposed to light.

The role of ethylene and methyl jasmonate in the mechanism of anthocyanin accumulation on the abaxial side of detached leaves of *K. blossfeldiana* exposed to light remains unexplained. It is generally known that ethylene, in relation to jasmonates, can interact synergistically. Ethylene inhibits processes induced by jasmonates, and jasmonates suppress physiological and biochemical processes induced by ethylene [30,31,32,35,36,37].

In *K. blossfeldiana* leaves taken directly from the growing plant, the content of almost all anthocyanins was higher on the adaxial side of the leaves compared to the abaxial side. In detached leaves kept with the adaxial side exposed to light for five days, the content of cyanidin di-glucoside and an unknown cyanidin derivative was higher on the abaxial side than on the adaxial side. On the other hand, the contents of cyanidin mono- and tri-glycosides and delphinidin on the adaxial and abaxial sides were similar to those in leaves taken directly from the plant.

In another experiment, it was observed that when the adaxial side of the detached leaf was removed and the abaxial part was exposed to light, anthocyanins accumulated similarly to those in the whole leaf. When the abaxial side was cut off along with almost all the parenchyma tissue, anthocyanin accumulation on the adaxial side was inhibited. Thus, it seems that anthocyanin accumulation on the abaxial side of a leaf is independent of its adaxial side.

## 3. Materials and Methods

### 3.1. Plant Materials

Plants of *Kalanchoë blossfeldiana* grown in a greenhouse at the National Institute of Horticultural Research in Skierniewice, Poland, were used. The shoot cuttings were taken for rooting in a mixture of soil, peat moss, and sand (2:1:1) in a greenhouse under natural conditions, then replanted separately in pots with the same growing medium. Leaves from three-month-old plants of *K. blossfeldiana* were used for the experiments. For the first experiment, large leaves (older), 40 mm in width, and small (younger) leaves, 25 mm in width, were used. The leaves were kept in a normal (adaxial side exposed to light) or inverted position (abaxial side exposed to light) in the greenhouse at temperatures ranging from 20 to 24 °C under ambient light conditions of 30–50 μmol/m^2^/s PPFD. Supplemental Figure S1 describes the method used in the experiments.

For the second experiment, only large leaves were used. In this experiment, detached leaves of *K. blossfeldiana* were treated with JA-Me at concentrations of 0.1 and 0.5% in lanolin paste. JA-Me was applied in the middle part of the leaf blade as a 1–2 mm-wide strip on its lower (abaxial) side. These leaves were kept in an inverted position (abaxial side exposed to light) in the greenhouse at 20–24 °C for five days. Control leaves were untreated or treated with lanolin only, and they were kept under the same conditions. All experiments were repeated three to five times, using 20 to 25 leaves per treatment.

### 3.2. Anthocyanin Extraction and Analysis

Anthocyanin extraction and analysis were performed using a modified method, as described by Wiczkowski et al. [46]. Freeze-dried and powdered leaves of *K. blossfeldiana* (0.1 g) were extracted with 0.4% *tri*-fluoro acetic acid (TFA) in methanol by vortexing (30 s) and sonication (30 s) (VC 750, Sonics & Materials, New Town, CT, USA). The solutions were centrifuged for 20 min (13,200× *g*, 4 °C, 5415R, Eppendorf, Hamburg, Germany), and the supernatants were combined into 5 mL volumetric flasks. This step was repeated five times. The volume was brought up to 5 mL with distilled water. After dilution and centrifugation, extracts (in triplicate) were analyzed by micro-HPLC-MS/MS-TOF. The samples were analyzed using an LC-200 Eksigent HPLC system coupled with a Triple TOF 5600^+^ mass spectrometer (AB SCIEX, Vaughan, ON, Canada). Chromatographic separation of anthocyanins after injecting 5 µL of sample solution was carried out on a HALO C18 column (2.7 µm, 100 × 0.5 mm, Eksigent, Vaughan, ON, Canada) with a flow rate of 15 µL/min at a temperature of 45 °C. Elution was carried out using a solvent gradient system consisting of solvent A (0.95% formic acid aqueous solution) and solvent B (0.95% formic acid in acetonitrile). The gradient was as follows: 0–0.3 min: 3% B, 0.3–2 min: 3–90% B, 2–4.5 min: 90% B, 4.5–4.7 min: 90–3% B, 4.5–5.0–3 min: 3% B. The analysis was conducted by scanning in positive ionization mode, with the following optimal conditions: ion spray voltage floating (ISVF): 5500 V, temperature: 350 °C, nebulizing gas (GS1): 35 psi, heater gas (GS2): 35 psi, curtain gas: 25 psi. The MS functioned in full-scan TOF-MS (100–2000 m/z) and MS/MS mode (70–1000 m/z). The declustering potential (DP) and collision energy (CE) for the full-scan MS experiment were 90 V and 10 eV, respectively, while for the MS/MS mode, they were 80 V and 40 eV, respectively. The collision energy spread (CES) entered was 15 eV. These parameters were optimized and introduced based on previous experience in analysis using external standards. Further, to control the quality of the analyses, complementary analyses of mixtures of external standards were performed at the beginning, middle, and end of the analysis series. The results of these analyses confirmed the absence of significant changes in signal intensity and accuracy.

Anthocyanin identification was based on a comparison of their retention time and MS/MS fragmentation spectrum (m/z values) with data from standards analysis, published sources, or/and on the interpretation of the fragmentation spectrum obtained. Quantitative determination of anthocyanins was carried out based on external standards using a pseudomolecular ion and a main fragmentation ion. The calibration curve (ranging from 0.2 to 3 µM, respectively) was linear with a correlation coefficient of 0.998.

Analyses were performed in three replicates. Analysis of variance (one-way ANOVA) and Tukey’s post hoc test were used to check the significance of differences between mean results.

### 3.3. Histological Analyses of K. blossfeldiana Leaves

Images of the abaxial side of three-month-old leaves of *K. blossfeldiana*, as well as their cross-sections, were prepared using a razor blade. Pictures of the preparations were taken using an Eclipse 80i light microscope (Nikon, Tokyo, Japan).

## 4. Conclusions

The present study shows that the leaves of *Kalanchoë blossfeldiana* contain a 15-component set of anthocyanins consisting of monoglycosides, diglycosides, triglycosides, their acyl derivatives, and four unknown derivatives of petunidin, cyanidin, and delphinidin. The positions in which the leaves were kept after being detached from the plant caused different responses of distinct types of anthocyanins. Among the anthocyanins present in *K. blossfeldiana*, delphinidin-rhamnoside-glucoside, cyanidin-rhamnoside-glucoside, and cyanidin-glucoside-glucoside were responsive to the stress caused by detaching the leaves and keeping them for five days with the abaxial side exposed to light. It is very likely that these anthocyanins accumulate under the epidermis of the abaxial side to protect it from excessive light, and their presence causes the visible red coloration of the leaves. The leaf positioning arrangement did not affect the contents of mono-glycosides, tri-glycosides, and acyl derivatives of cyanidin and delphinidin. The effect of leaf positioning was ambiguous for the unknown derivatives of petunidin, cyanidin, and delphinidin.

Application of methyl jasmonate (JA-Me) reduced the contents of delphinidin-rhamnoside-glucoside, cyanidin-rhamnoside-glucoside, and cyanidin-glucoside-glucoside but did not affect the contents of mono-glycosides and tri-glycosides. This effect is probably due to the presence of the aforementioned di-glycosides in the sub-epidermal cells of *K. blossfeldiana* leaves. These are directly exposed to JA-Me, leading to the inhibition of their biosynthesis and/or accumulation. The lack of a significant effect of JA-Me on the mono- and tri-glycosides of cyanidin and delphinidin may in turn indicate the presence of these anthocyanins in the mesophyll, as shown in the microscopic cross-sectional picture of the leaf.

The metabolic response in detached *K. blossfeldiana* leaves, involving changes in anthocyanin composition and accumulation sites, is likely a combined effect of light stress and wounding.

## Figures and Tables

**Table 1:** List of anthocyanins found in the leaves of *K. blossfeldiana* and their mass spectral data.

Anthocyanin	[M]^+^ (m/z)	MS/MS (m/z)
Delphinidin-rhamnoside	449	303
Delphinidin-rhamnoside-glucoside	611	449/303
Cyanidin-glucoside-glucoside	611	449/287
Cyanidin-rhamnoside	433	287
Cyanidin-rhamnoside-glucoside	595	433/287
Cyanidin-rhamnoside-rhamnoside-glucoside	741	579/433/287
Delphinidin-(coumaroyl)-glucoside-rhamnoside	757	449/303
Delphinidin derivative 1	473	303
Cyanidin derivative 1	484	287
Petunidin derivative 1	487	317
Petunidin derivative 2	501	317
Cyanidin-rhamnoside-rhamnoside	579	433/287
Delphinidin-(feruloyl)-rhamnoside	625	449/303
Delphinidin-(feruloyl)-glucoside	641	465/303
Cyanidin-rhamnoside-rhamnoside-xyloside	711	579/433/287

**Table 2:** Content (mg/g DW) of some anthocyanins in *K. blossfeldiana* leaves on the plant and after detachment and five-day storage in natural position (adaxial side exposed to light) or inverted position (abaxial side exposed to light). Description of anthocyanins: 1—cyanidin rhamnoside; 2—delphinidin rhamnoside; 3—cyanidin rhamnoside-glucoside-glucoside; 4—cyanidin rhamnoside-rhamnoside-xyloside; 5—delphinidin-(feruloyl)-rhamnoside; 6—delphinidin-(coumaroyl)-glucoside-rhamnoside; 7—delphinidin-(feruloyl)-glucoside; 8—delphinidin derivative 1; 9—cyanidin derivative 1; 10—petunidin derivative 1; 11—petunidin derivative 2. Mean results ± SD followed by the same letters do not differ significantly at *p* = 0.05.

Antho-cyanin	Large (Older) Leaves			Small (Younger) Leaves		
	On Plant	Detached and Kept in Natural Position	Detached and Kept in Inverted Position	On Plant	Detached and Kept in Natural Position	Detached and Kept in Inverted Position
1	0.334 ± 0.008 ^a^	0.279 ± 0.006 ^b^	0.261 ± 0.004 ^b^	0.343 ± 0.007 ^a^	0.323 ± 0.006 ^a^	0.278 ± 0.008 ^b^
2	0.151 ± 0.002 ^a^	0.144 ± 0.003 ^a^	0.142 ± 0.002 ^a^	0.144 ± 0.003 ^a^	0.145 ± 0.002 ^a^	0.144 ± 0.003 ^a^
3	0.112 ± 0.001 ^a^	0.105 ± 0.002 ^b^	0.092 ± 0.002 ^c^	0.115 ± 0.002 ^a^	0.102 ± 0.002 ^b^	0.104 ± 0.002 ^b^
4	0.034 ± 0.002 ^a^	0.032 ± 0.002 ^a^	0.028 ± 0.002 ^a^	0.031 ± 0.001 ^a^	0.033 ± 0.001 ^a^	0.032 ± 0.001 ^a^
5	0.021 ± 0.001 ^a^	0.016 ± 0.002 ^a^	0.021 ± 0.001 ^a^	0.024 ± 0.002 ^a^	0.024 ± 0.002 ^a^	0.026 ± 0.002 ^a^
6	0.052 ± 0.002 ^a^	0.051 ± 0.001 ^a^	0.048 ± 0.002 ^a^	0.046 ± 0.002 ^a^	0.049 ± 0.002 ^a^	0.044 ± 0.003 ^a^
7	0.018 ± 0.001 ^a^	0.019 ± 0.001 ^a^	0.020 ± 0.001 ^a^	0.022 ± 0.002 ^a^	0.022 ± 0.002 ^a^	0.023 ± 0.002 ^a^
8	0.114 ± 0.002 ^d^	0.090 ± 0.001 ^e^	0.094 ± 0.002 ^e^	0.155 ± 0.004 ^a^	0.134 ± 0.001 ^b^	0.129 ± 0.002 ^c^
9	0.071 ± 0.002 ^a^	0.063 ± 0.002 ^a^	0.042 ± 0.001 ^b^	0.036 ± 0.001 ^c^	0.017 ± 0.001 ^d^	0.018 ± 0.001 ^d^
10	2.896 ± 0.056 ^a^	2.396 ± 0.086 ^bc^	2.206 ± 0.038 ^c^	3.058 ± 0.071 ^a^	3.022 ± 0.060 ^a^	2.578 ± 0.065 ^b^
11	0.170 ± 0.003 ^b^	0.174 ± 0.002 ^b^	0.150 ± 0.001 ^c^	0.210 ± 0.003 ^a^	0.164 ± 0.004 ^b^	0.183 ± 0.004 ^b^

**Table 3:** Content (mg/g DW) of some anthocyanins in *K. blossfeldiana* leaves on the plant and detached, treated or untreated with methyl jasmonate (JA-Me), and stored for five days under ambient conditions. Description of anthocyanins: 1—cyanidin rhamnoside; 2—delphinidin rhamnoside; 3—cyanidin rhamnoside-glucoside-glucoside; 4—cyanidin rhamnoside-rhamnoside-xyloside; 5—delphinidin-(feruloyl)-rhamnoside; 6—delphinidin-(coumaroyl)-glucoside-rhamnoside; 7—delphinidin-(feruloyl)-glucoside; 8—delphinidin derivative 1; 9—cyanidin derivative 1; 10—petunidin derivative 1; 11—petunidin derivative 2. Mean results ± SD followed by the same letters do not differ significantly at *p* = 0.05.

Antho-cyanin	Leaves on Plant	Leaves Detached and Kept in Natural Position	Leaves Detached and Kept in Inverted Position			
			Not Treated	Treated with Lanolin	Treated with 0.5% MJ in Lanolin	Treated with 0.1% MJ in Lanolin
1	0.432 ± 0.004 ^a^	0.375 ± 0.006 ^b^	0.348 ± 0.004 ^c^	0.392 ± 0.008 ^b^	0.388 ± 0.008 ^b^	0.383 ± 0.009 ^b^
2	0.213 ± 0.004 ^a^	0.175 ± 0.005 ^b^	0.158 ± 0.004 ^b^	0.171 ± 0.005 ^b^	0.167 ± 0.003 ^b^	0.176 ± 0.004 ^b^
3	0.141 ± 0.002 ^a^	0.133 ± 0.003 ^a^	0.132 ± 0.003 ^a^	0.150 ± 0.006 ^a^	0.132 ± 0.004 ^a^	0.124 ± 0.006 ^a^
4	0.034 ± 0.002 ^a^	0.031 ± 0.002 ^a^	0.035 ± 0.002 ^a^	0.038 ± 0.003 ^a^	0.035 ± 0.002 ^a^	0.037 ± 0.002 ^a^
5	0.021 ± 0.001 ^a^	0.025 ± 0.002 ^a^	0.021 ± 0.002 ^a^	0.024 ± 0.001 ^a^	0.017 ± 0.002 ^a^	0.019 ± 0.002 ^a^
6	0.066 ± 0.003 ^a^	0.057 ± 0.002 ^a^	0.054 ± 0.002 ^a^	0.064 ± 0.003 ^a^	0.054 ± 0.002 ^a^	0.056 ± 0.002 ^a^
7	0.022 ± 0.001 ^a^	0.017 ± 0.003 ^a^	0.020 ± 0.002 ^a^	0.024 ± 0.002 ^a^	0.016 ± 0.002 ^a^	0.017 ± 0.002 ^a^
8	0.094 ± 0.002 ^a^	0.073 ± 0.002 ^b^	0.076 ± 0.002 ^b^	0.094 ± 0.002 ^a^	0.078 ± 0.002 ^b^	0.077 ± 0.002 ^b^
9	0.049 ± 0.002 ^ab^	0.033 ± 0.002 ^c^	0.048 ± 0.002 ^ab^	0.061 ± 0.003 ^a^	0.047 ± 0.001 ^b^	0.042 ± 0.002 ^bc^
10	2.199 ± 0.088 ^ab^	1.502 ± 0.068 ^c^	2.104 ± 0.080 ^ab^	2.396 ± 0.038 ^a^	1.872 ± 0.060 ^b^	1.936 ± 0.035 ^b^
11	0.158 ± 0.004 ^bc^	0.122 ± 0.003 ^d^	0.155 ± 0.004 ^b^	0.175 ± 0.003 ^ab^	0.132 ± 0.002 ^cd^	0.143 ± 0.003 ^c^

## Data Availability

The data presented in this study are available in this article.

## Supplementary Materials

The supporting information can be downloaded at: https://www.mdpi.com/article/10.3390/ijms25052875/s1.
