# Supplementary material for: Exposure to Light of the Abaxial versus Adaxial Side of Detached Kalanchoë blossfeldiana Leaves Affects Anthocyanin Content and Composition Differently
Source: Int J Mol Sci. 2024 Mar 1;25(5):2875. doi: 10.3390/ijms25052875 (PMC10932424; doi:10.3390/ijms25052875)
Supplement: Supplementary file 1 [file ijms-25-02875-s001.zip › ijms-2884132-supplementary.pdf]

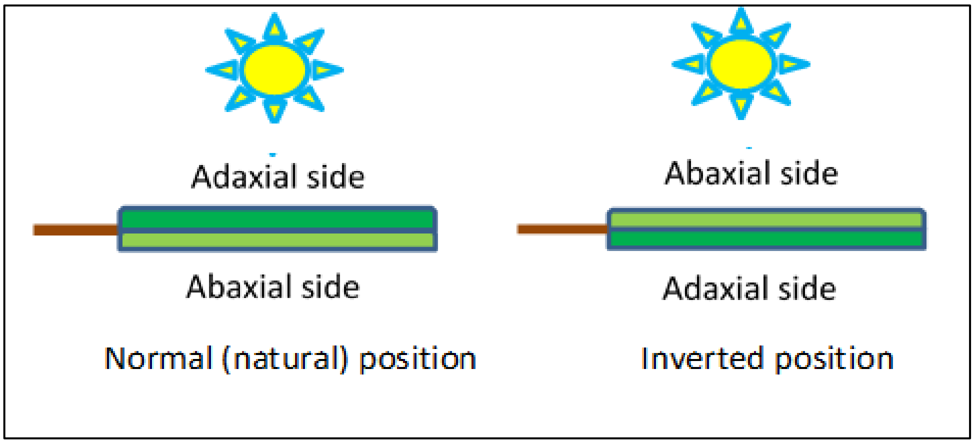

Figure S1. Diagram for conducting an experiment on the effect of 5-day keeping of *K. blossfeldiana* leaves after they are detached from the plant
